# Correction: Impact of nucleic acid and methylated H3K9 binding activities of Suv39h1 on its heterochromatin assembly

**DOI:** 10.7554/eLife.31641

**Published:** 2017-09-06

**Authors:** Atsuko Shirai, Takayuki Kawaguchi, Hideaki Shimojo, Daisuke Muramatsu, Mayumi Ishida-Yonetani, Yoshifumi Nishimura, Hiroshi Kimura, Jun-ichi Nakayama, Yoichi Shinkai

Shirai A, Kawaguchi T, Shimojo H, Muramatsu D, Ishida-Yonetani M, Nishimura Y, Kimura H, Nakayama J, Shinkai Y. 2017. Impact of nucleic acid and methylated H3K9 binding activities of Suv39h1 on its heterochromatin assembly. *eLife* **6**:e25317. doi: 10.7554/eLife.25317.

Published 1, August 2017

Due to an error introduced during the production process, the image for Figure 1 – figure supplement 1 was a duplicate of the image for Figure 2 – figure supplement 1.

The correct image is shown here:

**Figure 1:**
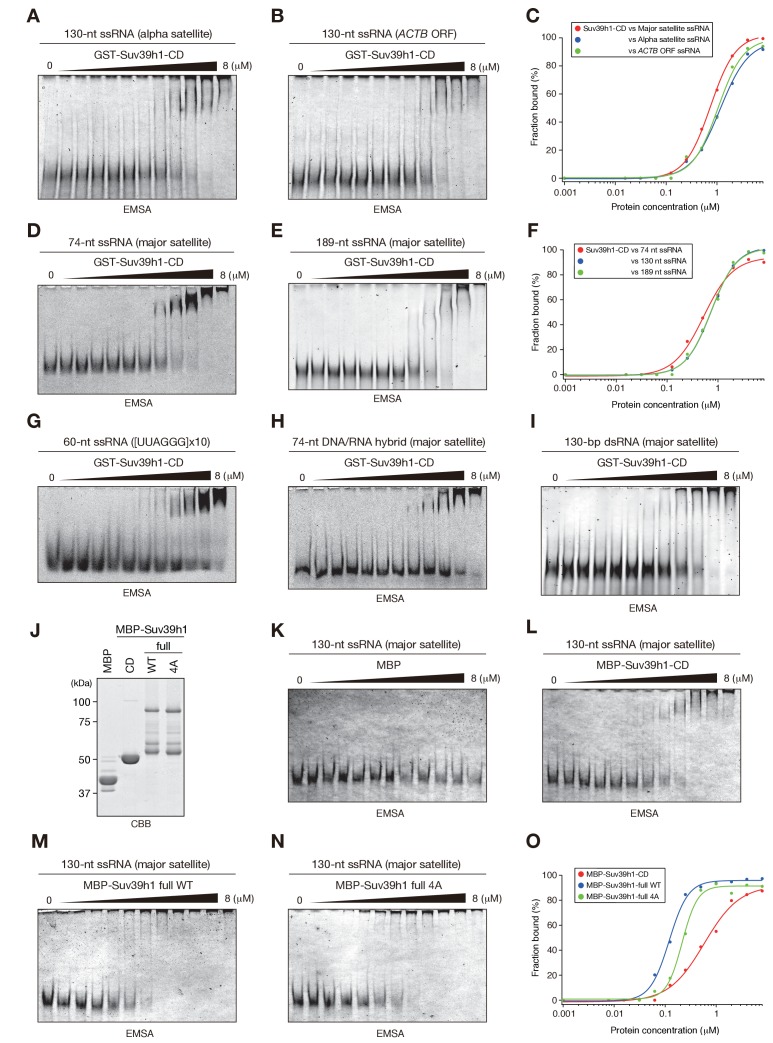


The incorrect duplicated image is shown here for reference:

**Figure 2:**
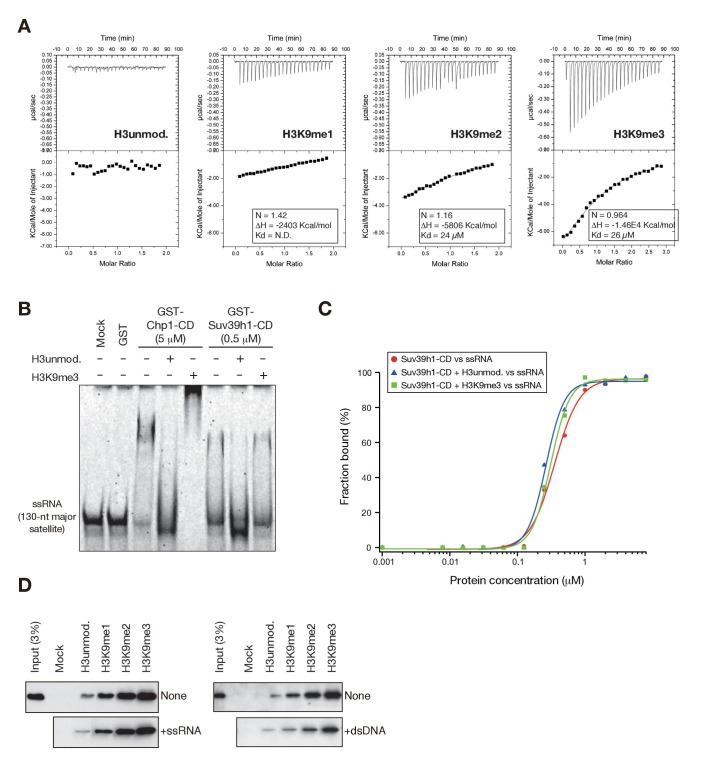


The article has been corrected accordingly.

